# Tropism and Infectivity of Pandemic Influenza A H1N1/09 Virus in the Human Placenta

**DOI:** 10.3390/v14122807

**Published:** 2022-12-15

**Authors:** Yan-Na Xiao, Fei-Yuan Yu, Qian Xu, Jiang Gu

**Affiliations:** 1 Provincial Key Laboratory of Infectious Diseases and Molecular Immunopathology, Shantou University Medical College, Shantou 515041, China; 2 Department of Cell Biology and Genetics, Shantou University Medical College, Shantou 515041, China; 3 Jinxin Research Institute for Reproductive Medicine and Genetics, Chengdu 610066, China

**Keywords:** influenza A virus, pdmH1N1, human placenta

## Abstract

Influenza virus infection in pregnant women may put the fetus at higher risk; however, to date, there has been no detailed research about the expression of influenza virus receptors in the human placenta. We employed the lectin staining technique, which is a classic influenza virus receptor research method for studying the distribution of viral receptors in the human placenta. In addition, we examined the susceptibility of the human placenta to H1N1/09, by detecting viral proteins and RNA at different time points post-infection. We found that the human placenta expressed both avian and human influenza A virus receptors (α-2, 3-linked sialic acid and α-2, 6-linked sialic acid). In addition, H1N1/09 did not only infect the human placenta, but also replicated and was released into the culture media. We concluded that the human placenta is susceptible to the 2009 influenza A virus (H1N1/09) infection, and that particular attention should be paid to shielding pregnant women from infection during influenza season.

## 1. Introduction

Research shows that pregnant women face a higher risk of infection than the general population during flu outbreaks. According to previous studies, there was a higher rate of miscarriage for pregnant women exposed to and infected by the 1918 pandemic influenza virus (subtype H1N1) during the Spanish flu period [1,2]. Following the Asian influenza outbreak of 1957–58, it was reported that adverse outcomes of pregnancy, such as neonate congenital malformation, spontaneous abortion, pre-term delivery and fetal demise, were increased [3,4,5]. In 2007, influenza A virus genes were detected in placental trophoblasts and in the respiratory tracts, during autopsies of fetuses whose mothers had been infected by H5N1 [6]. Lieberman et al. reported a case of second trimester fetal death, which occurred after the mother was exposed to seasonal influenza A (H1N1): in that case, the influenza virus was detected in the placenta tissue, by immunohistochemical staining [7].

The 2009 influenza A (H1N1/09) virus originated in North America in April 2009, spread rapidly throughout the world in the following months, and has been circulating seasonally since the pandemic [8]. Recent investigations have reported poor outcomes for pregnant women infected with H1N1/09 [9]. Studies have shown that H1N1 infection in pregnancy can lead to an increased risk of adverse pregnancy outcomes, such as spontaneous abortion, premature delivery, fetal distress and stillbirth [9,10,11]. Cetinkaya M. et al. reported an H1N1/09-positive premature infant, born at 32 weeks of gestation, whose mother had a confirmed H1N1/09 infection diagnosed by real-time reverse transcriptase polymerase chain reaction (rRT-PCR) [12]. In addition, H1N1/09 was found in the thoracic aorta of a mouse model. The above evidence suggests that the H1N1/09 virus can be transmitted across the placenta barrier. It is generally believed that sialic acid (SA) is the receptor responsible for the influenza A virus infection [13]. Avian influenza viruses (AIVs) and human influenza viruses (HuIVs) bind to different sialyloligosaccharides: the former binds preferentially to SA linked to galactose by α-2, 3 linkage (SAα-2, 3-Gal), while the latter prefers to bind to SA linked to galactose by α-2, 6 linkage (SAα-2, 6-Gal) [14,15]. However, the distribution of influenza virus receptors in the human placenta, the tropism of the influenza A virus to the human placenta and the kinetics of H1N1/09 in the human placenta and placental cell lines have not been investigated.

In this study, we aimed to examine the distribution of influenza A viral receptors in the human placenta, and the possible reactions of the human placenta and placental cell lines to the influenza A virus H1N1/09 infection.

## 2. Materials and Methods

### 2.1. Cell Lines and Ex Vivo Tissue Samples

JAR cells (human choriocarcinoma) were obtained from ATCC (Manassas, VA, USA). TEV-1 is a human first trimester extravillous trophoblast cell line (provided by Professor George Tsai, Hong Kong University). Cell culture was performed in accordance with ATCC instructions. The JAR cell line was cultured in RPMI-1640 (Cat# C22400500BT, Gibco, New York, NY, USA) containing 10% fetal bovine serum (Cat# 10099-141, Gibco, NY, USA) and 1% penicillin–streptomycin (Cat# 15140-122, Gibco, NY, USA). The TEV-1 cell line was cultured in DMEM-F12 (Cat# C11330500BT, Gibco, NY, USA) containing 10% fetal bovine serum and 1% penicillin–streptomycin. Both the cell lines were cultured at 37 °C in a humidified atmosphere with 5% CO_2_.

Human placenta tissue was obtained at affiliated hospitals of Shantou University Medical College, and was approved by The Ethics Review Committee of Shantou University Medical College (approval number: SUMC-2021-42; date of approval: 6 May 2021).

### 2.2. Virus

The influenza A H1N1/009 virus strain, A/Nanchang/8002/2009 (H1N1), was kindly provided by Professor David Kelvin (Shantou University Medical College), and was propagated in 9-day-old embryonic chicken eggs. Viral titers were checked with the 50% Tissue Culture Infectious Dose (TCID_50_) and 50% Egg Infections Dose (EID_50_) assay, before inoculation.

### 2.3. Lectin Staining

The following biotinylated sialic acid-specific lectins were used to detect the avian and human influenza receptors [16]: biotinylated Maackia amurensis lectin I (MAA I); biotinylated Maackia amurensis lectin II (MAA II); and biotinylated Sambucus nigra agglutinin (SNA). MAA I (Cat# B-1315) and MAA II (Cat# B-1265) are markers for avian influenza virus receptors (AIV-R), while SNA (Cat# B-1305) is the marker for human influenza virus receptors (HuIV-R); the lectins were purchased from Vector Laboratories (Burlingame, CA, USA). Lectin histochemistry was performed, as described previously [17]. In brief, formalin-fixed, paraffin-embedded tissue sections were deparaffinized, immersed in 3% hydrogen peroxide and incubated with 5% bovine serum albumin, to block nonspecific staining. The tissue sections were then allowed to incubate overnight with SNA (1.5 μg/mL) and MAA (6 μg/mL) in buffer at 4 °C. An SABC kit (Dako, Carpinteria, CA, USA) was used to optimize the contrast between specific labeling and background. Biotinylated lectin binding was revealed by a 3,3′-diaminobenzidine tetrahydrochloride (DAB) substrate-chromogen kit (Zymed Labs, South San Francisco, CA, USA), which produced a brown color, and the slides were counterstained with hematoxylin. Omission of the lectins was used as a negative control.

### 2.4. Neuraminidase Pretreatment

In order to ensure the specificity of the lectin staining, we digested the sialic acid receptors, ahead of the lectin staining, with neuraminidase (NA) (Cat# P0720S, New England BioLabs, MA, USA). In brief, following deparaffinization and immersion in 3% hydrogen peroxide, the slides were incubated with 5 U/uL of NA for 24 h (h) at 37 °C. The slides were subsequently blocked with 5% BSA. The lectin staining was then performed, as described above. Additional negative controls included incubation with PBS instead of NA.

### 2.5. Immunohistochemistry (IHC)

In order to identify positive cell types of lectin staining in human placenta, we performed double staining. In brief, paraffin-embedded tissue sections were deparaffinized and immersed in 3% hydrogen peroxide, to eliminate endogenous peroxidase activity. Antigen retrieval was performed, by heating the tissue sections at 96°C in 0.01 mol/L citrate buffer (pH 6.0) for 20 min (min). Monoclonal antibodies to the following cell markers were used: CD68 (Cat# ZM-0060, ZSGB-BIO, Beijing, China) (macrophages); placental alkaline phosphatase (PLAP; Cat# ZA-0513, ZSGB-BIO, Beijing, China) (syncytiotrophoblast); E-cadherin (Cat# ZM-0092, ZSGB-BIO, Beijing, China) (cytotrophoblast); and CD34 (Cat# ZA-0550, ZSGB-BIO, Beijing, China) (endothelial cells). After incubation with a primary antibody at 4 °C overnight, the tissue sections were incubated with PV9000 IHC kit (Cat# PV9000, ZSGB-BIO, Beijing, China), following the kit instructions. To visualize specific signals, 3-Amino-9-ethylcarbazole (AEC) (Cat# 01-12, GBI Labs, Rockville, MA, USA) was used, which gave a brown–red reaction color. The slides were counterstained with hematoxylin. The controls for IHC included omission of the primary antibodies, and pre-absorption tests in which the primary antibodies were pre-incubated with the corresponding antigens, before immunostaining.

### 2.6. Stain–Decolorize–Stain (SDS) Method

In order to identify the target cells of the influenza A virus in the human placenta, we also performed the SDS method. In brief, after staining of the lectin by AEC, photos of the positive fields in the tissue slide were taken; then, the cover slides were removed in a 60 °C water bath, followed by color removal with 80% ethanol washing, till the lectin staining completely disappeared. The tissue slides were subsequently heated to 95 °C in citrate buffer, to block antigen–antibody crossing interaction; then, it was incubated with different kinds of cell markers overnight at 4 °C. Following washing in PBS, the slides were stained, by following the instructions of the SAP 9100 kit (Cat# SAP-9100, Zymed Labs, South San Francisco, CA, USA). The color was developed with Nitroblue tetrazolium/5-bromo-4-chloro-3-indolyl phosphate (NBT/BCIP; Cat# S3771, Promega, Madison, WI, USA), which produces a purple–black color. The same fields of the tissue sections were photographed again. The position stainings of photos taken at exactly the same locations were assigned different colors, and were superimposed on one another, to compare the distributions of different antigens on the same tissue section.

### 2.7. Viral Infection of Human Placental Cell Lines

Cells cultured in 12-well plates were washed with phosphate-buffered saline (PBS), and were infected with the influenza A/Nanchang/8002/2009 (H1N1) at a multiplicity of infection (MOI) of 2 for 2 h at 37 °C. After a 2 h absorption, the inocula were removed, and the cells were incubated with medium supplemented with TPCK-treated trypsin (Cat# T1426, Sigma–Aldrich, St. Louis, MO, USA) and BSA, for predetermined time periods. Negative control was mock-infected. The supernatants were collected at 2, 6, 12, 18 and 24 h post-infection, for determination of viral titer by TCID_50_, while the infected cells were harvested at the same time points, to detect the relative quantity of influenza A virus M2 genes with real-time reverse-transcription polymerase chain reaction (RT-PCR) and antigen expression, by immunocytochemistry (ICC) and Western blot.

### 2.8. Viral Infection of Ex Vivo Tissues

Fresh human placenta tissue was collected at delivery, and was used within 1–2 h of collection. The ex vivo tissue specimens were washed with culture medium (F-12K nutrient mixture (Cat# 21127022, Gibco, NY, USA) with 15% FBS, L-glutamine and antibiotics), and were incubated with the virus at 1 × 10^7^ TCID_50_/_mL_ for placenta tissue at 37 °C in the medium; after 2 h, the uncombined virus was washed away, and the infected tissue was sustained for 2, 24 and 48 h post-infection. The supernatants were collected for the detection of viral titer at different time points, by the TCID_50_ method. The ex vivo tissue was harvested, for detection of the relative quantity of influenza A virus M2 genes with real-time reverse-transcription polymerase chain reaction (rRT-PCR) and antigen expression with immunohistochemistry (IHC).

### 2.9. Detection of H1N1/09 Influenza A Virus Antigen with ICC/IHC Techniques

JAR and TEV-1 cells, grown on glass coverslips in 12-well plates, were fixed with 4% paraformaldehyde for 30 min, washed with PBS three times, then blocked with normal goat serum for 1 h at room temperature, and incubated with mouse anti-H1N1 hemagglutinin (HA) antibody (Cat# IT-003-SW, Immune Technology, New York, NY, USA) overnight at 4 °C. After washing with PBS, the cells were incubated with PV9000, by following the kit instructions. To visualize specific signals, AEC was used, by following the kit instructions. The cells were counterstained with hematoxylin. Omission of the primary antibody and mocked infection of cell lines were used as negative controls.

The harvested tissue was fixed with 4% paraformaldehyde, and was processed for 0.5 routine paraffin embedding and histochemical analysis with the mouse anti-H1N1 HA antibody (Immune Technology, USA), as reported previously [18]. Omission of the primary antibody and mock infection of ex vivo tissue were used as the negative controls.

### 2.10. Detection of H1N1/09 Influenza A Virus Antigen with Western Blot

We examined the cell lysate with immunoblotting, to assay for the presence of H1N1/09 nucleoprotein (NP). The cells cultured in 6-well plates were washed once with 2 mL of ice-cold PBS, harvested in 150 µL of RIPA lysis buffer (150 mM NaCl, 50 mM Tris-HCl [pH 8], 1 mM EDTA [pH 8], 1% NP-40, 0.1% SDS and 0.1% deoxycholate with 1 tablet of protease inhibitor cocktail [Roche] per 10 mL buffer), sonicated for 10 s and centrifuged at 13,000× *g* at 4 °C for 20 min. The protein concentration of the cellular lysates was determined with the BCA Assay (Cat# 23225, Pierce, Rockford, IL, USA). Protein aliquots were electrophoresed on 10% SDS-PAGE gels, and were transferred to nitrocellulose Protran membranes (Cat# 10402495, Whatman, Dassel, Germany). The blots were incubated for 1 hr at room temperature in blocking buffer (5% skim milk in TBST [150 mM NaCl, 10 mM Tris (pH 8), 0.1% Tween-20]), incubated with the mouse anti-influenza A virus NP antibody (Cat# GTX125989, Genetex, Irvine, CA, USA) overnight, at a dilution of 1:3000 in blocking buffer, washed four times in TBST for 5 min each, incubated with a fluorescence-labeled secondary antibody at 1:5000 in blocking buffer for 1 h at room temperature and washed four times in TBST for 5 min each. Signals were detected with the odyssey system (Gene Company, *S*an Diego, CA, USA). Human β-actin was used as an internal control.

### 2.11. Real-Time PCR

RNA was extracted from the cell lines and ex vivo tissue, at the indicated time points post-infection, with Trizol (Cat# 15596-018, Invitrogen, Carlsbad, CA, USA), by following the manufacturer’s instructions. The RNA was transcribed to cDNA by High-Capacity cDNA Reverse Transcription Kits (Cat# 4368813, Applied Biosystem, Foster City, CA, USA). The expressions of influenza A virus genes in infected cell lines and ex vivo tissue were examined by relative quantity real-time PCR performed with specific primers targeting the influenza A virus M2 gene. Data were analyzed with 2^-ΔΔCT^ method and the mean values were normalized by β-actin gene expression. SYBR ^®^ Premix Ex Taq II (Cat# DRR820B, TaKaRa, Beijing, China) was used in the reaction, and primer pairs are provided in Table 1.

### 2.12. TCID_50_ Assay

The supernatants of the H1N1/09-infected placental cell lines and the ex vivo placenta were assayed for viable virus in the MDCK cells, by TCID_50_ assay. A 10-fold serial dilution of the supernatants was added to the cultured MDCK cells (in a 96-well plate), and incubated for 2 h; then, the supernatants were removed from the cells, and replaced by DMEM containing TPCK-treated trypsin and BSA. The cells were cultured for 6 days, at which time the cell culture supernatants were tested for viable virus by a haemagglutination assay, using 0.5% (*v*/*v*) prepared chicken red blood cells, as previously described [19]. The Muench and Reed method was used to calculate TCID_50_. Mock-infected groups were used as negative controls. Viral store (EID_50_ = 10^7.3^) was used as a positive control.

### 2.13. Statistics Analysis

Statistical analyses were performed using GraphPad Prism 5. The resulting *p*-value < 0.05, using the two-tailed unpaired t-test, was considered statistically significant.

## 3. Results

### 3.1. Distribution of Influenza A Viral Receptors in Human Placenta Tissue

**Figure 1:**
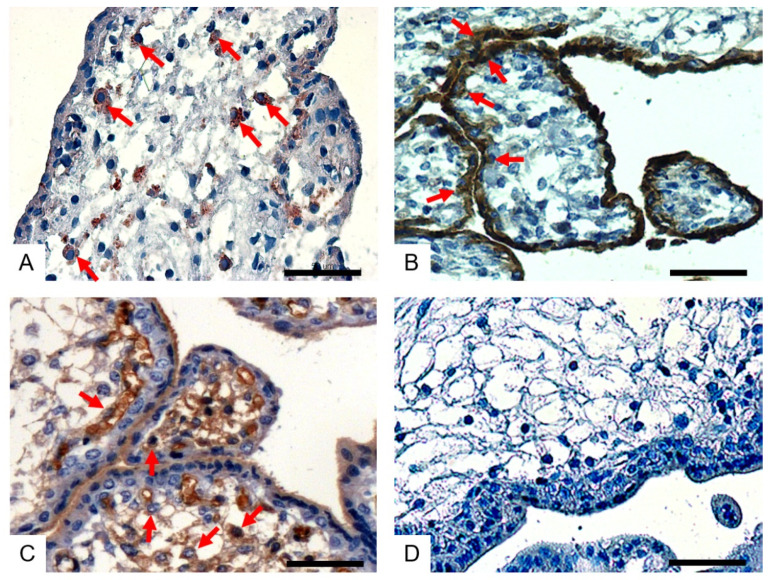
The distribution of AIV-Rs and HuIV-Rs in the human placenta. The placenta tissue samples were stained with biotinylated Sambucus nigra agglutinin (SNA, for HuIV-R) (**A**), biotinylated Maackia amurensis lectin I (MAA I, for AIV-R) (**B**) and biotinylated Maackia amurensis lectin II (MAA II, for AIV-R) (**C**). A section incubated with PBS was used as a negative control (**D**). All sections were counterstained with hematoxylin. The arrows indicate positive cells. Scale bar = 50 μM.

In order to investigate the expression of influenza virus receptors in the placenta, lectin staining was performed. We found that SNA (Figure 1A), MAA I (Figure 1B) and MAA II (Figure 1C) were all positive in the placenta tissue, but that their corresponding positive cells were different. These results indicate that the human placenta expresses both human and avian influenza virus receptors, but that the distribution of these two receptors is different.

**Figure 2:**
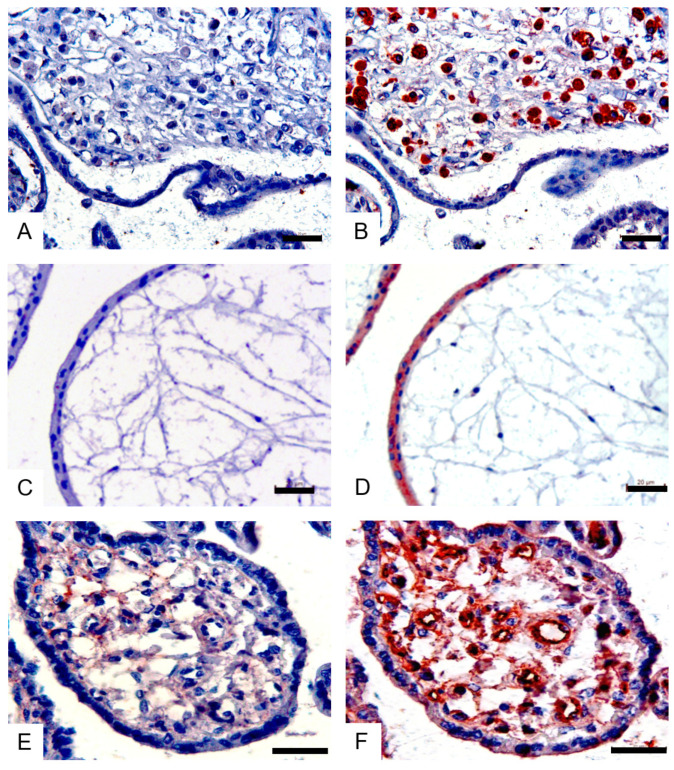
NA pretreatment lectin staining of human placenta tissue. Serial sections of the placenta tissue were stained with SNA (**A**,**B**), MAA I (**C**,**D**) and MAA II (**E**,**F**), after being treated with (**A**,**C**,**E**) or without (**B**,**D**,**F**) neuraminidase. All sections were counterstained with hematoxylin. Scale bar = 20 μm.

Neuraminidase (NA), also known as sialidase, cleaves sialic acid. In order to clarify the specific staining of the lectin (Figure 1), neuraminidase was used to treat the placenta tissue, and it was found that the SNA, MAA I and MAA II staining were negative (Figure 2A, Figure 2C and Figure 2E, respectively); however, Lectin staining was positive in serial sections of the corresponding placenta tissue without neuraminidase treatment (Figure 2B,D,F), which further demonstrated that both human influenza virus receptors and avian influenza virus receptors are distributed in the human placenta.

**Figure 3:**
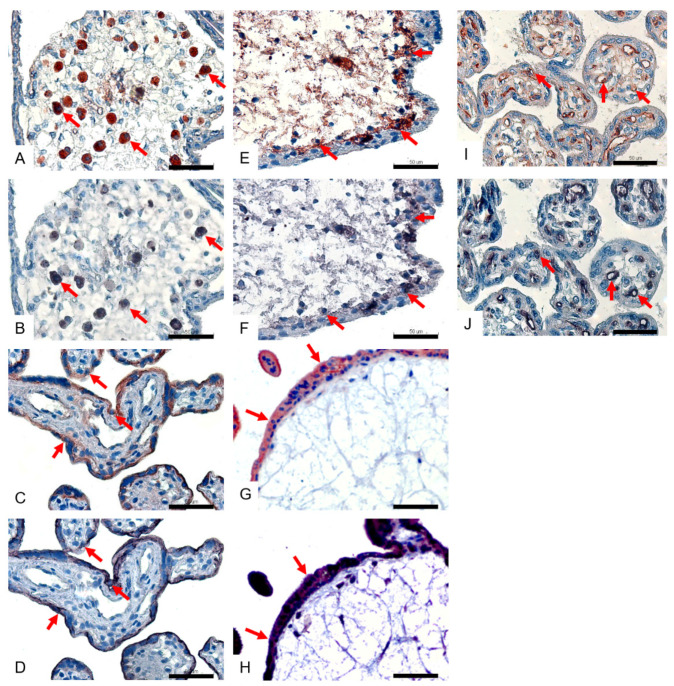
Confirmation of lectin-positive cell types by special IHC in a human placenta. Brown and brown–red colors stand for the positive signals in the lectin staining (**A**,**C**,**E**,**G**,**I**), and the purple–black color stands for positive signals in cell marker immunohistochemistry (**B**,**D**,**F**,**H**,**J**). In the Stain–Decolorize–Stain (SDS) method, the macrophages (red arrow) were positive for SNA (**A**) and CD68 (macrophage marker) (**B**); the syncytiotrophoblasts (red arrow) were positive for MAA I (**C**) and PLAP (syncytiotrophoblast marker) (**D**); the cytotrophoblasts (red arrow) were positive for MAA II (**E**) and E-cadherin (cytotrophoblast marker) (**F**); in consecutive sections, the syncytiotrophoblasts (red arrow) were positive for MAA I (**G**) and PLAP (syncytiotrophoblast marker) (**H**); the vascular endothelial cells (red arrow) were positive for MAA II (I) and CD34 (vascular endothelial cell marker) (**J**). All sections were counterstained with hematoxylin. Scale bar = 50 μm.

The SDS immunohistochemistry method was performed further, to identify the cell types of these positive cells. We found that the SNA-positive cells (Figure 3A) were macrophages (Hofbauer cells) (Figure 3B), that the MAA I-positive cells (Figure 3C) were syncytiotrophoblast cells (Figure 3D) and that the MAA II-positive cells (Figure 3E) were cytotrophoblast cells (Figure 3F). The results of the consecutive sections showed that the MAA I-positive cells were syncytiotrophoblast cells (Figure 3G,H) and that the MAA II-positive cells were vascular endothelial cells (Figure 3I,J).

### 3.2. Infection of H1N1/09 in Ex Vivo Placenta Tissue

**Figure 4:**
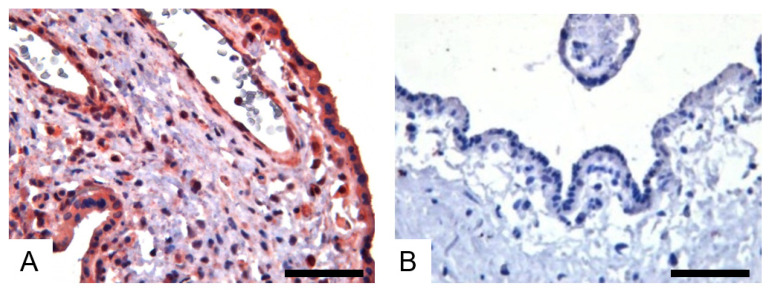
Detection of HA in H1N1/09-infected fresh placenta tissue with IHC. The brown–red color stands for the positive signals. Infection of human placenta (**A**) by H1N1/09. Mock infection of human placenta (**B**) was performed as control. All sections were counterstained with hematoxylin. Scale bar = 50 μm.

To study whether H1N1/09 can infect human tissue, we examined the expression of H1N1/09 HA protein in the infected ex vivo human placenta tissue at 24 h post-infection (h.p.i). The IHC results showed that H1N1/09 HA was widely distributed in the placenta, including the syncytiotrophoblast cells, cytotrophoblast cells, Haufbauer cells and vascular endothelial cells (Figure 4A), while no positive signal was detected in the mock-infected placenta tissue (Figure 4B).

### 3.3. Infection of H1N1/09 in Placenta Cell Lines

**Figure 5:**
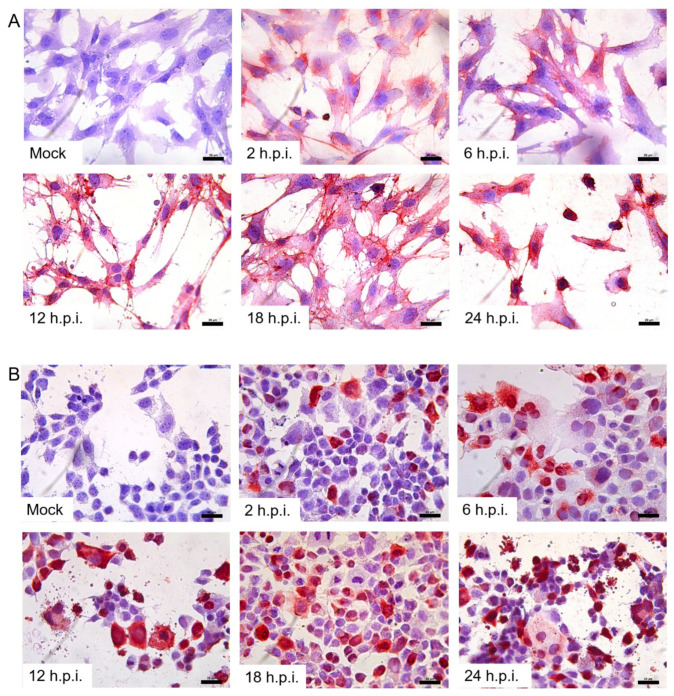
HA expression in H1N1/09-infected human placental cell lines. The brown–red color stands for the positive signals. An antibody against H1N1 HA was used to detect HA expression. TEV-1 cells (**A**) were mock-infected or infected with H1N1/09 for 2 h, 6 h, 12 h, 18 h and 24 h, respectively; JAR cells (**B**) were mock-infected or infected with H1N1/09 for 2 h, 6 h, 12 h, 18 h and 24 h, respectively. All cells were counterstained with hematoxylin. Scale bar = 20 μm.

To further confirm the infectivity of H1N1/09 in placenta cells, we examined the expression of H1N1/09 HA protein in the infected placenta cell lines at different time points post-infection with IHC. We found that both the TEV-1 and JAR cell lines were positive for H1N1/09 HA protein (Figure 5A,B), compared to the mock-infected cells. In addition, as the infection periods were prolonged, from 2 h.p.i. 6 h.p.i, 12 h.p.i. 18 h.p.i. to 24 h.p.i., the amount of HA protein became more abundant in the above two cell lines.

### 3.4. H1N1/09 Nucleoprotein (NP) and the M2 Gene Were Detected in Placenta Cells

**Figure 6:**
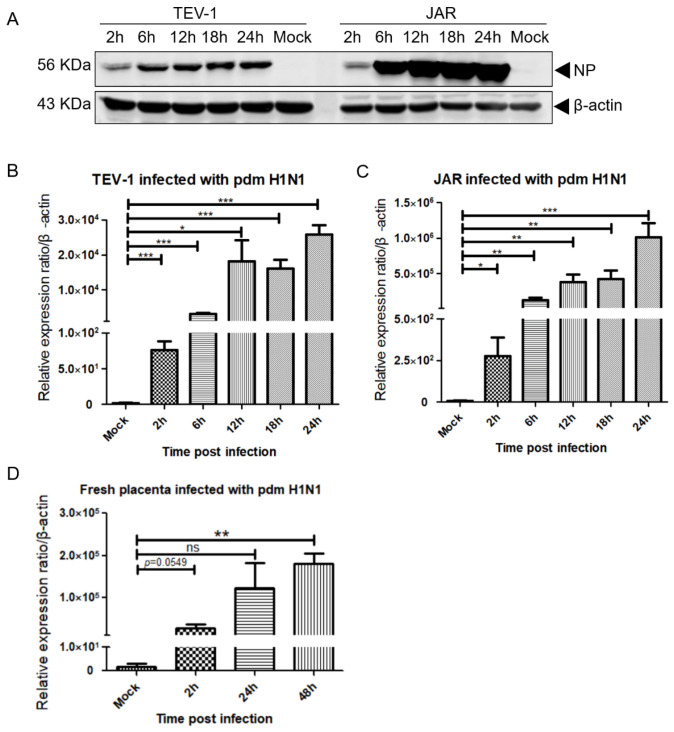
The detection of NP protein and viral M2 RNA in an H1N1/09-infected human placenta. A mouse anti-influenza A virus NP antibody was used to detect the nucleoprotein, and a β-actin antibody was used as an internal control (**A**). Quantification of viral M2 RNA in H1N1/09-infected TEV-1 (**B**), JAR (**C**) cells and placenta tissue (**D**), using real-time PCR. Data are presented as mean ± SEM, n = 3–6, * *p*< 0.05, ** <0.01, *** <0.001 versus mock-infected; ns, non-significant.

In the placental cell lines infected with the H1N1/09 virus, we found that the HA antigen increased with the duration of infection. In addition, we found that the amount of nucleoprotein (NP) also increased as the post-infection period became longer in the H1N1/09-infected cell lysate (Figure 6A). We hypothesized that H1N1/09 is not only capable of infecting, but also of replicating, in placenta cells. Influenza virus replication is reflected by influenza matrix gene expression [20]. In order to verify the above hypothesis, we examined the expression of influenza A virus matrix 2 (M2) RNA in H1N1/09-infected placenta cells and ex vivo placenta tissue. From 2 h.p.i. to 24 h.p.i, there was an increase of the M2 RNA as the post-infection period became longer in the TEV-1 cells (Figure 6B) and the JAR cells (Figure 6C). The placenta homogenate had the same tendency, as the post-infection duration became longer: the fold change of the M2 RNA became more numerous compared to the mock infection (Figure 6D). The above results also showed that both the NP and the M2 in the JAR cells were more abundant than that in the TEV-1 cells (Figure 6A–C).

### 3.5. H1N1/09 Virus Release from Infected Human Placenta Tissue

In order to study whether there were influenza virions released from the infected human placenta tissue, we used the TCID_50_ method to detect viable viruses in the supernatant. The store virus H1N1/09 (EID_50_ = 10^7.3^) was used as a positive control; the mock-infected group was used as a negative control. The results showed that the viral titer of the store virus was 10^5.3^, and at the time points of 2 h.p.i., 24 h.p.i., 48 h.p.i. and 72 h.p.i., the viral titer was 0, 10^2.1^, 10^4.2^ and 10^3.6^, respectively (Table 2). The release of the viruses appeared to peak at 48 h post-infection.

## 4. Discussion

In the present study, we used Sambucus nigra agglutinin (SNA) and two isoforms of Maackia amurensis agglutinin (MAA) to detect influenza virus receptors. SNA identifies SAα2-6Gal, and MAA I is specific for SAα2-3Galβ1-4GalNAc, while MAA II is more specific toward SAα2-3Galβ1-3GalNAc [16]. We found that SNA, MAA I and MAA II were all positive in the human placenta tissue, which indicates that both AIV-Rs and HuIV-Rs are expressed in the human placenta.

There are four reports of suspected H1N1/09 vertical transmission in newborns whose mothers were infected [10]. Vasquez et al. reported a case of a premature baby boy delivered by cesarean section due to fetal distress [21]. The mother was infected with H1N1 during pregnancy. The newborn, who had no contact with the mother after birth, was subsequently identified as being infected with the H1N1 influenza virus [21]; however, the placenta was not tested for virology and pathology, so direct evidence of vertical transmission was lacking. Similar reports have been made in Thailand [22], India [23] and Turkey [12]. These cases suggest that HIN1/09 can be transmitted vertically; however, it is not clear exactly how the influenza viruses were transmitted. In this study, we studied the distribution of influenza receptors in the placenta, by lectin staining, revealing the possibility of the vertical transmission of the influenza virus through receptors in the placenta. We found that HuIV-Rs were mainly located in Haufbauer cells, which was in keeping with the report of Yao et al. [18]; AIV-Rs were mainly in syncytiotrophoblasts, cytotrophoblasts, and vascular endothelial cells, which was not in accordance with the report of Yao et al—a difference which may have been attributable to the lectin used for the AIV-Rs detection: the previous study only used MAA II, rather than both MAA I and MAA II, as in the current study [18].

Regarding the expression of both HuIV-Rs and AIV-Rs in the human placenta, we proposed that the influenza A virus is prone to infecting the human placenta; therefore, we further investigated the susceptibility and kinetics of the Pandemic H1N1/09 influenza virus in the human placenta and placental cell lines, through detection of H1N1/09 RNA and proteins. The results showed that H1N1/09 was not only able to infect but also to replicate in ex vivo placenta tissue and placental cell lines (TEV-1 and JAR). As the post-infection duration was prolonged, more viruses appeared to have reproduced. Liong et al. found that no viral messenger RNA was found in the placenta tissue of H1N1/09-infected pregnant mice in vivo; however, they also found that influenza A virus segment 3 polymerase (PA) was in the maternal thoracic aorta [24], indicating influenza A virus dissemination to major maternal blood vessels. In addition, we could detect the virus in the supernatant of H1N1/09-infected human placenta tissue at different time points. At 48 h post-infection, the viruses released into the medium reached a peak; however, we did not detect released viruses in the supernatants of the placental cell lines, which was in agreement with the report of Shihoko et al. [25], who did not detect viral particles in the supernatant of the trophoblast cell line (H8 and Sw.7). The quantity of released viruses may possibly have been below the detectable threshold, by the method of TCID_50_, or perhaps no infectious progeny viruses were assembled in these placental cell lines [26]; moreover, the HA protein of H1N1/09 was widely expressed in the human placenta, including syncytiotrophoblasts, cytotrophoblasts, vascular endothelial cells and Haufbauer cells. The distribution of HA expression was in agreement with that of MAA I and MAA II. Previous studies have shown that H1N1/09 viruses can also bind to α-2, 3-linked sialic acids [27,28]: this indicates that both the AIV-Rs and the HuIV-Rs distributed in the placenta tissue were permissible for H1N1/09 viral infection. Based on the results of real-time PCR and WB, the susceptibility of the JAR cell line was much stronger than that of the TEV-1 cell line, which indicates that villous trophoblasts may be more vulnerable to H1N1/09 infection than extravillous trophoblasts. The infection of trophoblasts may contribute to the spontaneous abortion of infected pregnant patients.

## 5. Conclusions

Taken together, our findings suggest that the human placenta expresses both human and avian influenza virus receptors, but that there is a difference in their distribution patterns. The H1N1/09 influenza virus can infect target cells through both receptors. We also found that H1N1/09 is capable of not only infecting but also of replicating in the placenta, the mechanism of which may contribute to influenza A virus vertical transmission.

## Figures and Tables

**Table 1:** Oligonucleotide sequence of the primers.

Primers	Sequence 5′-3′
M2 Forward	AAGACCAATCCTGTCACCTCTGA
M2 Reverse	CAAAGCGTCTACGCTGCAGTCC
β-actin Forward	ATGGGTCAGAAGGATTCCTATGTG
β-actin Reverse	CTTCATGAGGTAGTCAGTCAGGTC

**Table 2:** Viral titer of the supernatant of the placenta tissue culture, using the method of TCID_50._

	Time Post-Infection			
Mock	2 h	24 h	48 h	72 h
0	0	10^2.1^	10^4.2^	10^3.6^

## Data Availability

Not applicable.
